# Identification of epicatechin as the bioactive constituent in polyphenol enriched extracts that demonstrate a beneficial effect on allergic symptoms

**DOI:** 10.1186/2045-7022-3-S3-P7

**Published:** 2013-07-25

**Authors:** A Singh, A Demont, L Actis Goretta, A Lévêques, S Holvoet, S Nutten

**Affiliations:** 1 Nestlé Research Center, Lausanne, Switzerland

## Background

Polyphenols are naturally derived bioactive compounds that have been attributed numerous health benefits. We have previously characterized the beneficial effect of a polyphenol enriched apple extract in a murine model of food allergy. The objective of the current study was to elucidate the class of bioactive polyphenols that are implicated in the beneficial anti-allergic effect of polyphenol enriched extracts.

## Methods

In a well established murine model of food allergy, BALB/c mice that were sensitized to ovalbumin were administered polyphenol enriched extracts or purified epicatechin polyphenol in their diet for 8 days after the last sensitization. Sensitized mice were challenged orally with ovalbumin following treatment with polyphenol enriched extracts or purified epicatechin and allergic symptoms were compared between control and treated mice, in addition to allergen-specific serum immunoglobulins and gene expression profiles in the intestine.

## Results

Polyphenol enriched apple extracts demonstrated a differentiating ability *in vivo* to modulate allergic symptoms. Interestingly, the beneficial effect could be linked to polyphenol content of the extracts. The anti-allergy effect can be attributed to the epicatechin polyphenol, as oral administration of purified epicatechin in the diet of sensitized mice modulated allergic symptoms in a dose dependant manner. Immune parameters were also impacted via epicatechin administration.

## Conclusion

Epicatechin is a key bioactive polyphenol that demonstrates anti-allergy properties.

## Disclosure of interest

None declared.

